# Risk factor analysis of perioperative complications in patients with rheumatoid arthritis undergoing primary cervical spine surgery

**DOI:** 10.1186/s13075-022-02767-0

**Published:** 2022-03-31

**Authors:** Koji Sakuraba, Yuki Omori, Kazuhiro Kai, Kazumasa Terada, Nobuo Kobara, Satoshi Kamura, Kenjiro Fujimura, Hirofumi Bekki, Masanari Ohta, Hisa-aki Miyahara, Jun-ichi Fukushi

**Affiliations:** 1 Department of Orthopaedic Surgery and Rheumatology, National Hospital Organization Kyushu Medical Center, Jigyohama 1-8-1, Chuo-ku, Fukuoka, 810-8563 Japan; 2 Clinical Research Center, National Hospital Organization Kyushu Medical Center, Jigyohama 1-8-1, Chuo-ku, Fukuoka, 810-8563 Japan

**Keywords:** Rheumatoid arthritis, Cervical spine surgery, Perioperative complications, Occipito-cervical/thoracic fusion, Occipito-cervical fusion, Cervical spine lesion, Subaxial subluxation, ASA-PS, Prednisolone

## Abstract

**Background:**

Rheumatoid arthritis (RA) often causes cervical spine lesions as the disease condition progresses, which induce occipital neuralgia or cervical myelopathy requiring surgical interventions. Meanwhile, patients with RA are susceptible to infection or other complications in the perioperative period because they frequently have comorbidities and use immunosuppressive medications. However, the risk factors or characteristics of patients with RA who experience perioperative complications after cervical spine surgery remain unknown. A risk factor analysis of perioperative complications in patients with RA who underwent primary cervical spine surgery was conducted in the present study.

**Methods:**

A total of 139 patients with RA who underwent primary cervical spine surgery from January 2001 to March 2020 were retrospectively investigated. Age and height, weight, serum albumin, serum C-reactive protein, American Society of Anesthesiologists Physical Status (ASA-PS), Charlson comorbidity index, medications used, cervical spine lesion, surgery time, bleeding volume, and procedures were collected from medical records to compare the patients with complications to those without complications after surgery. The risk factors for perioperative complications were assessed by univariate and multivariate logistic regression analysis.

**Results:**

Twenty-eight patients (20.1%) had perioperative complications. Perioperative complications were significantly associated with the following factors [data presented as odds ratio]: lower height [0.928, p=0.007], higher ASA-PS [2.296, *p*=0.048], longer operation time [1.013, *p*=0.003], more bleeding volume [1.004, *p*=0.04], higher rates of vertical subluxation [2.914, *p*=0.015] and subaxial subluxation (SAS) [2.507, *p*=0.036], occipito-cervical (OC) fusion [3.438, *p*=0.023], and occipito-cervical/thoracic (long) fusion [8.021, *p*=0.002] in univariate analyses. In multivariate analyses, lower height [0.915, *p*=0.005], higher ASA-PS [2.622, *p*=0.045] and long fusion [7.289, *p*=0.008] remained risk factors. High-dose prednisolone use [1.247, *p*=0.028], SAS [6.413, *p*=0.018], OC fusion [17.93, *p*=0.034], and long fusion [108.1, *p*<0.001] were associated with severe complications.

**Conclusions:**

ASA-PS and long fusion could be indicators predicting perioperative complications in patients with RA after cervical spine surgery. In addition, cervical spine lesions requiring OC fusion or long fusion and high-dose prednisolone use were suggested to be risk factors for increasing severe complications.

## Background

Rheumatoid arthritis (RA) is a systemic inflammatory disease that induces not only destructive arthritis in the systemic joint but also distinct spine lesions [1–3]. Surgical interventions are often required as these musculoskeletal disabilities progress [3–6]. In the cervical spine, RA-related spine lesions can cause occipital neuralgia and myelopathy, which are major issues that interfere with activities of daily living [7, 8]. Surgical interventions are mandatory to relieve neck pain, to improve physical function, and to decrease the risk of mortality, when patients do not improve with conservative treatments [9–11].

RA-derived systemic inflammation leads to spinal pathology through bony erosion and ligamentous laxity [12–16]. The prevalence of cervical spine lesions has been reported across a wide range, 9% to 88%, of patients with RA who have neck pain [3, 8, 13]. In addition, five percent of patients with cervical spine lesions because of RA had observable neurological deficits [7]. Although surgical intervention has been the mainstay of treatment to resolve robust occipital pain and neurological deficits, perioperative complications, including infections, are of concern because patients with RA are more likely to have comorbidities and take immunosuppressive agents, such as biologics and JAK inhibitors. In various retrospective cohort series, the incidence of perioperative complications was reported to range from 8 to 30% [17–20]. However, the risk of perioperative complications after cervical spine surgery in patients with RA has not been well established.

The purpose of this study was to clarify the risk factors for perioperative complications in consecutive patients with RA who underwent cervical spine surgery at one institution. Demographic status, RA medication, type of cervical spine lesion, and surgery-related factors were retrospectively investigated. In addition, we evaluated comorbidities at the time of surgery that might affect the occurrence of complications using the Charlson comorbidity index (CCI) [21] and American Society of Anesthesiologists Physical Status (ASA-PS) [22, 23] to assess preoperative physical condition.

## Patients and methods

From January 2001 to March 2020, 139 patients with RA underwent primary cervical spine surgery in our institution because of cervical myelopathy or occipital neuralgia. All these 139 cases were included in the present study. We reviewed medical records during a series of hospitalizations that underwent surgery and confirmed any perioperative complications. Physical information (sex, age, height, and weight), disease history of RA including medication used, laboratory data (serum albumin and serum C-reactive protein), comorbidities, radiographs to clarify the cervical spine lesion with RA before surgery, and surgical procedure were collected. In accordance with Common Terminology Criteria for Adverse Event (CTCAE) version 5 [24], which displays grades 1 through 5 with unique clinical descriptions of severity for each adverse event based on the general guidelines, severe complications were defined as grade 3, which is severe or medically significant but not immediately life-threatening (hospitalization or prolongation of hospitalization indicated, disabling, or limiting selfcare ADLs) or higher grades. All patients fulfilled the American Rheumatism Association 1987 revised criteria [25] or the 2010 American College of Rheumatology/European League Against Rheumatism classification criteria [26] for RA.

### Evaluation of comorbidities

Comorbidities were evaluated by the CCI and ASA-PS. The CCI is a weighted index to predict short-term and long-term outcomes, including mortality rates, by assessing comorbidity levels by taking into account both the number and severity of 19 predefined comorbid conditions and age [21]. The score can range from 0 to 37. The ASA-PS classification system was used to assess and communicate patients’ medical comorbidities before anesthesia; patients are classified into six classes based on the type of comorbidities, lifestyle factors such as smoking and alcohol consumption, and physical condition, including body mass index and the status of respiration and circulation: class I, normal health; class II, mild systemic disease; class III, severe systemic disease; class IV, severe systemic disease that is a constant threat to life; class V, moribund patients who are not expected to survive without the operation; and class VI, a declared brain-dead patient [22, 23].

### Cervical spine lesion

**Fig. 1:**
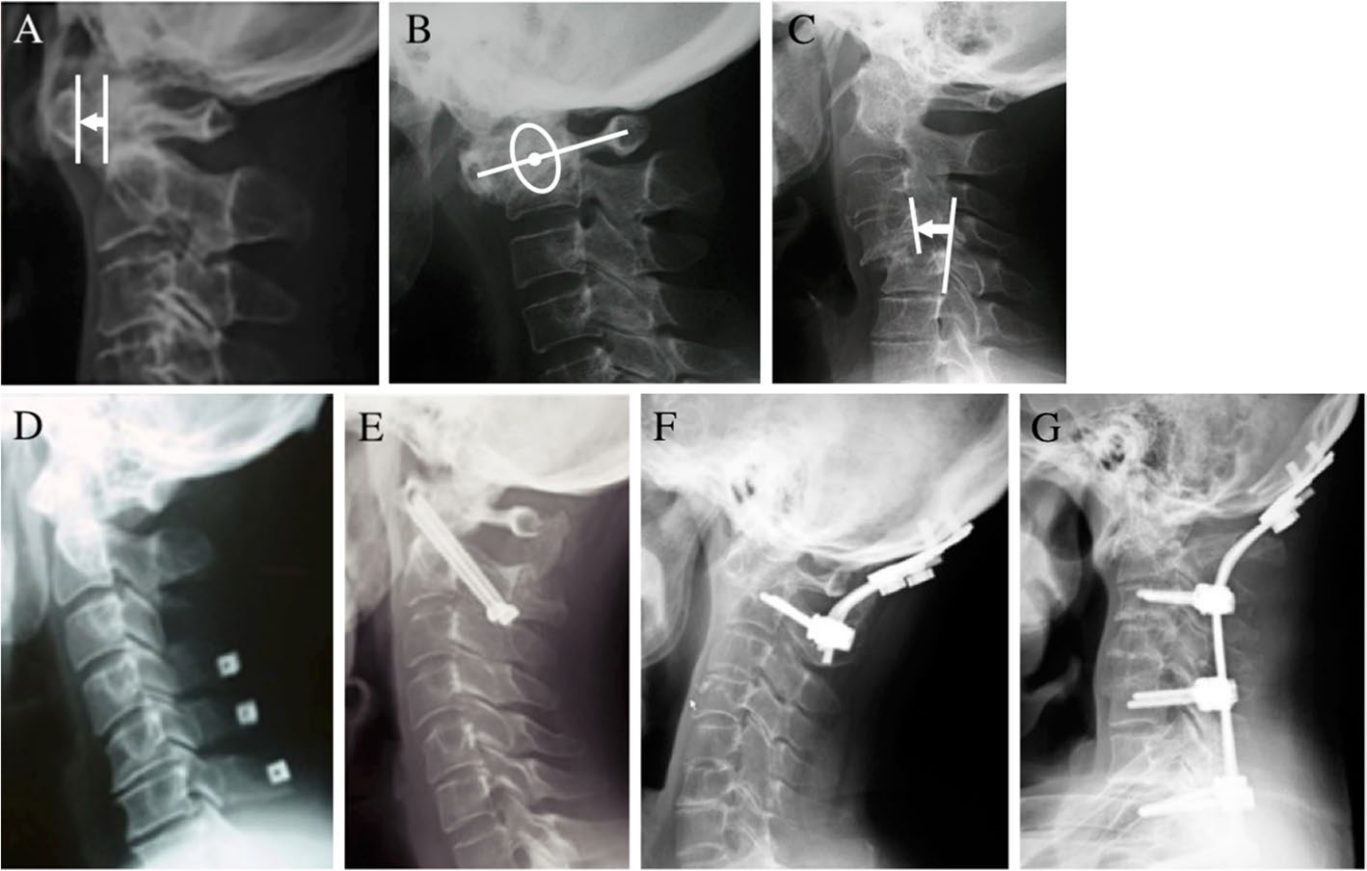
Cervical lesions associated with RA and surgical procedures for individual cervical lesions. **A** Atlantoaxial subluxation (AAS) was defined as an expansion of the atlantodental interval over 3 mm (between the white lines) at the flexed position on X-ray lateral view. **B** Vertical subluxation (VS) was defined as a shortening of the Ranawat C1–C2 index to less than 15 mm for men and 13 mm for women. The measurement of the Ranawat index was made from the center of the pedicles of the axis (white circle and dot) to a line connecting the midpoint of the anterior and posterior arches of the atlas (white line). **C** Subaxial subluxation (SAS) was diagnosed as migration of the superior vertebra compared to the inferior vertebra over 3 mm. Migration distance was measured between posterior walls of adjacent vertebrae (between the lines). **D**–**G** Four types of surgical procedures were performed for cervical spine lesions in patients with RA. Laminoplasty was performed for myelopathy in the stable cervical spine (**D**). Fixative procedures were chosen depending on how unstable the cervical spine was: atlantoaxial fusion for unstable AAS (**E**), occipital-cervical fusion for VS with or without AAS (**F**), and occipital/thoracic fusion for SAS that included AAS and/or VS (**G**)

Cervical spine lesions with RA were classified into three types by radiographic imaging (Fig. 1). Atlantoaxial subluxation (AAS) was defined as an expansion of the atlantodental interval (ADI) of more than 3 mm in the flexed position (Fig. 1A) [3]. The ADI was measured from the posterior edge of the anterior arch of the atlas to the anterior edge of the axis dens in the lateral view. Vertical subluxation (VS) was assessed by the Ranawat C1-C2 index [3, 16]. The measurement of the Ranawat index was made from the center of the pedicles of the axis to a line connecting the midpoint of the anterior and posterior arches of the atlas. Anything less than 15 mm for men and 13 mm for women confirmed VS (Fig. 1B). Subaxial subluxation (SAS) was diagnosed as migration of more than 3 mm from the superior vertebra compared to the inferior vertebra [3]. Migration distance was measured between posterior walls of adjacent vertebrae (Fig. 1C).

### Procedure

All surgical treatments were performed by two spine surgeons. Fixative procedures were performed due to occipital neuralgia and/or myelopathy based on the unstable alignment of the cervical spine. Unstable alignment was defined by deterioration of subluxation in the flexed position or reduceable cases in the extended position. VS with robust neck pain was also defined as unstable. The types of fixative procedures were chosen depending on the affected levels of the cervical spine. Atlantoaxial fusion (C1/2 fusion) was performed for unstable AAS (Fig. 1E). VS was stabilized in situ through posterior spine fixation with an occipito-cervical fusion (OC fusion) system (Fig. 1F). Since SAS was usually accompanied by AAS and VS, occipito-cervical/thoracic fusion (long fusion) was conducted (Fig. 1G). Laminoplasty was performed for myelopathy that was not associated with an unstable alignment of the cervical spine (Fig. 1D).

### Statistical analysis

Univariate and multivariate logistic regression analyses were performed for detecting risk factors for perioperative complications. To select the predictive variables for the multivariable analysis, stepwise regression analysis was applied. When there were variables closely related to each other, one of the variables was chosen to enter into stepwise analysis. All tests were two-tailed, and statistical significance was defined by a *p* value < 0.05. The analyses were conducted with JMP ver. 14 (SAS Institute Inc, NC, USA).

## Results

The baseline characteristics of the patients at the time of surgery are shown in Table 1. The mean age was 66.5 years, and the mean disease duration was 19.7 years. Regarding the evaluation of comorbidities, the mean ASA-PS score was 2.3, and the CCI score was 1.6. The proportion of patients taking oral prednisolone was high (84.9%), while that taking methotrexate was approximately 40%. Eighteen patients (13%) took biological agents or JAK inhibitors (infliximab, 1; etanercept, 6; adalimumab, 2; golimumab, 2; abatacept, 2; tocilizumab, 4; baricitinib, 1). Cervical spine lesions due to RA were found in 81.3%; AAS was observed in 66.2%; VS in 43.5%, and SAS in 30.0%. One-third of cervical spine lesions overlapped with each other.

**Table 1:** Baseline characteristics at the time of surgery

Items	Data
Patients (male/female), *n*	139 (35/104)
Age, *years*	66.5±1.0
Height, *m*	1.52±0.09
Weight, *kg*	51.1±11.6
BMI, *kg/m*^*2*^	21.9±3.9
BSA, *m*^*2*^	1.42±0.18
Serum albumin, *g/dl*	3.7±0.5
Charlson comorbidity index	1.60±0.8
ASA-PS	2.3±0.5
Disease duration, *years*	19.7±14.0
CRP, *mg/dl*	1.11±1.33
Medication	
Biologics/JAK inhibitor, *n* (%)	18 (13)
Methotrexate, *n* (%)	57 (41)
Prednisolone, *n* (%)	118 (84.9)
Cervical spine lesion	
Spondylosis, *n* (%)	26 (18.7)
AAS, *n* (%)	92 (66.2)
VS, *n* (%)	60 (43.5)
SAS, *n* (%)	41 (30.0)

Mean ± standard deviation (SD)

*BMI* body mass index, *CRP* C-reactive protein, *ASA-PS* American Society of Anesthesiologists Physical Status, *AAS* atlantoaxial subluxation, *VS* vertical subluxation, *SAS* subaxial subluxation

Of the 139 patients who underwent cervical spine surgery, 28 patients (20.1%) experienced perioperative complications (Table 2). Two patients experienced two complications: one had prolonged delirium and delayed wound healing, and the other had separate instances of dural injury and SSI. One-third of the complications were defined as severe (*n*=10, 33%), and those were more likely to occur in patients with OC fusion and long fusion (Table 2). Infectious complications were the most common complication (40%, *n*=12), and half of them were SSIs (Tables 2 and 3). There was no significant difference in the incidence of infection with or without the administration of prednisolone, methotrexate, or biologics and JAK inhibitors (Table 3). SSIs also showed no difference based on taking or not taking any particular type of medication (Table 3). There were two patients who died within 90 days after surgery: one died of acute deterioration of interstitial pneumonia at twelve weeks after laminoplasty, and the other died of pneumonia at 6 weeks after OC fusion.

**Table 2:** Perioperative complications of cervical spine surgery in patients with RA

	Total (*n*=139)	Laminoplasty (*n*=63)	C1/2 fusion (*n*=33)	OC fusion (*n*=30)	Long fusion (*n*=13)
Severe, *n* (%)	10 (7.19)	1 (0.72)	1 (0.72)	3 (2.16)	5 (6.95)
Airway constriction	2			1	1
Acute deterioration of interstitial pneumonia	1	1			
Acute myocardial infarction	1				1
Postoperative hyponatremia	1				1
Pneumonia	3		1	2	
Prolonged severe delirium	1				1
Upper gastrointestinal bleeding	1				1
Mild/Moderate, *n* (%)	20 (14.4)	8 (5.76)	2 (1.49)	8 (5.76)	2 (1.49)
Anaphylactic shock	1		1		
C5 palsy	2	2			
Delayed wound healing	3	2			1
Delirium	1	1			
Dural injury	3	1		2	
Herpes zoster	1			1	
SSI	6	1	1	3	1
UTI	1	1			
Skin infection	2			2	

*C1/2 fusion* atlantoaxial fusion, *OC fusion* occipito-cervical fusion, *SSI* surgical site infection, *UTI* urinary tract infection

**Table 3:** Associations between medications and perioperative infectious complications of cervical spine surgery in patients with RA (*n*=12)

	Total (139)	Infection (12)	*p* value	SSI (6)	*p* value
Biologics/JAK inhibitor, *n* (%)	18	2 (11.1)	.688	1 (5.56)	.789
w/o Biologics/JAK inhibitor, *n* (%)	121	10 (8.26)		5 (4.41)	
Methotrexate, *n* (%)	57	4 (7.02)	.572	2 (3.51)	.693
w/o Methotrexate, *n* (%)	82	8 (9.76)		4 (4.89)	
Prednisolone, *n* (%)	118	10 (8.47)	.875	6 (5.1)	.156
w/o Prednisolone, *n* (%)	21	2 (9.52)		0 (0)	

*w/o* without, *SSI* surgical site infection

To identify the risk factors for perioperative complications, baseline characteristics and surgery-related factors were compared between patients with complications and those without complications (Table 4). In the univariate analyses, the patients with complications had significantly shorter height [*p*=0.007], higher ASA-PS [*p*=0.048], and higher prevalence of VS [*p*=0.015] and SAS [*p*=0.036]. Regarding operative factors, prolonged operation time [*p*=0.003], heavy bleeding [*p*=0.040], OC fusion [*p*=0.023, vs. laminoplasty] and long fusion [*p*=0.002, vs. laminoplasty] significantly increased the risk for complications (Table 4).

**Table 4:** Univariate logistic regression analysis of risk factors for all complications of cervical spine surgery in patients with RA

	No complication	Complication	Univariable	
	(111)	(28)	OR (95% CI)	*p* value
Age, *years*	66.1 ± 9.60	68.0 ± 11.4	1.019 (0.977–1.063)	.379
Sex, male/female, *n*	30/81	5/23	1.704 (0.594–4.888)	.322
Height, *m*	1.532 ± 0.089	1.479 ± 0.091	0.928 (0.880–0.980)	.007
Weight, *kg*	51.6 ± 11.1	49.2 ± 13.2	0.982 (0.946–1.019)	.327
BMI, *kg/m*^*2*^	21.8 ± 3.7	22.3 ± 4.7	1.029 (0.925–1.144)	.602
BSA, *m*^*2*^	1.43± 0.18	1.37 ± 0.21	0.128 (0.011–1.428)	.095
Serum albumin, *g/dl*	3.72 ± 0.48	3.60 ± 0.46	0.594 (0.247–1.428)	.243
Charlson comorbidity index	1.73 ± 1.02	1.68 ± 0.86	0.947 (0.614–1.460)	.806
ASA-PS	2.29 ± 0.49	2.50 ± 0.51	2.296 (1.007–5.235)	.048
Disease duration, *years*	20.2 ± 14.5	17.6 ± 12.1	0.986 (0.955–1.017)	.363
CRP, *mg/dl*	1.07 ± 1.20	1.12 ± 1.37	0.972 (0.707–1.337)	.862
Medication				
Biologics/JAK inhibitor, *n* (%)	13 (11.7)	5 (17.9)	1.639 (0.531–5.058)	.390
Methotrexate, *n* (%)	46 (41.4)	11 (39.3)	0.914 (0.392–2.133)	.836
Methotrexate, *mg*	2.67 ± 0.35	2.29 ± 0.59	0.969 (0.858–1.095)	.612
Prednisolone, *n* (%)	92 (82.9)	26 (92.9)	2.685 (0.587–12.28)	.203
Prednisolone, *mg*	4.79 ± 0.33	5.73 ± 0.52	1.083 (0.961–1.221)	.193
Cervical spine lesion				
AAS, *n* (%)	73 (65.8)	19 (67.9)	1.099 (0.454–2.662)	.834
VS, *n* (%)	42 (38.2)	18 (64.3)	2.914 (1.229–6.911)	.015
SAS, *n* (%)	28 (25.7)	13 (46.4)	2.507 (1.063–5.913)	.036
Operation time, *minutes*	134.3 ± 43.3	167.1 ± 62.4	1.013 (1.004–1.021)	.003
Bleeding volume, *g*	72.5 ± 79.2	124.0 ± 177.5	1.004 (1.000–1.007)	.040
Procedure				
Laminoplasty, *n* (%)	55 (49.6)	8 (28.6)	Ref	Ref
C1/2 fusion, *n* (%)	30 (27.0)	3 (10.7)	0.688 (0.170–2.787)	.600
OC fusion, *n* (%)	20 (18.0)	10 (35.7)	3.438 (1.189–9.934)	.023
Long fusion, *n* (%)	6 (5.4)	7 (25.0)	8.021 (2.145–29.99)	.002

Mean ± standard deviation (SD)

*OR* odds ratio, *CI* confidence interval, *Ref* reference, *BMI* body mass index, *CRP* C-reactive protein, *ASA-PS* American Society of Anesthesiologists Physical Status, *AAS* atlantoaxial subluxation, *VS* vertical subluxation, *SAS* subaxial subluxation, *C1/2 fusion* atlantoaxial fusion, *OC fusion* occipito-cervical fusion

Next, we further demonstrated multivariate analysis using risk factors for perioperative complications that were suggested to be significant in univariate analysis. However, cervical spine lesion by RA and selected procedure are supposed to be confounding factors to each other. We therefore made two models for multivariate analysis: Model 1 for cervical spine lesions and Model 2 for surgical procedures. In addition, stepwise regression analyses were demonstrated to identify appropriate candidates from all variables that were suggested as a significant risk factor by univariate analysis. Multivariate analysis including cervical spine lesions (Model 1) revealed that short height [*p*=0.005], high ASA-PS [*p*=0.045], and short duration of RA [*p*=0.012] were correlated with an increased risk for complications (Table 5). In the multivariate analysis including surgical procedures (Model 2), short height [*p*=0.034], short duration of RA [*p*=0.017], and performing long fusion [*p*=0.008] were shown to increase complications (Table 5).

**Table 5:** Multivariate logistic regression analysis of risk factors for all complications of cervical spine surgery in patients with RA

	Model 1		Model 2	
	OR (95% CI)	*p* value	OR (95% CI)	*p* value
Height	0.915 (0.860–0.974)	.005	0.931 (0.872–0.995)	.034
ASA-PS	2.622 (1.023–6.717)	.045	2.141 (0.817–5.611)	.123
Median duration of RA	0.953 (0.918–0.990)	.012	0.950 (0.911–0.991)	.017
Cervical spine lesion				
SAS	2.555 (0.993–6.571)	.052		
Procedure				
Laminoplasty			Ref	
C1/2 fusion			0.956 (0.221–4.132)	.952
OC fusion			2.467 (0.777–7.826)	.125
Long fusion			7.289 (1.694–31.36)	.008

Model 1: Multivariate analysis excluding procedure because of confounding factor of cervical spine lesion

Model 2: Multivariate analysis excluding cervical spine lesion because of confounding factor of procedure

*OR* odds ratio, *CI* confidence interval, *Ref* reference, *ASA-PS* American Society of Anesthesiologists Physical Status, *AAS* atlantoaxial subluxation, *VS* vertical subluxation, *SAS* subaxial subluxation, *C1/2 fusion* atlantoaxial fusion, *OC fusion* occipito-cervical fusion

One-third of all complications were severe cases (Table 2). We demonstrated additional multivariate analysis (Table 6) in the same way as demonstrated for all complications to detect the risk factor for severe complication. Multivariate analysis including cervical spine lesions (Model 3) revealed that the risk of severe complication significantly increased by high-dose administration of prednisolone [*p*=0.028] and the existence of SAS [*p*=0.018] (Table 6). Multivariate analysis including surgical procedures (Model 4) showed the risk of that was significantly increased by OC fusion [*p*=0.034] and long fusion [*p*<0.001] (Table 6).

**Table 6:** Multivariate logistic regression analysis of risk factors for severe complications of cervical spine surgery in patients with RA

	Model 3		Model 4	
	OR (95% CI)	*p* value	OR (95% CI)	*p* value
Height	<0.001 (<0.001–1.310)	.057		
PSL	1.247 (1.024–1.519)	.028	2.141 (0.817–5.611)	.072
Median duration of RA			0.950 (0.911–0.991)	.126
Cervical spine lesion				
SAS	6.413 (1.381–29.79)	.018		
Procedure				
Laminoplasty			Ref	
C1/2 fusion			2.919 (0.146–58.29)	.483
OC fusion			17.93 (1.242–258.8)	.034
Long fusion			108.1 (6.876–1699)	<.001

Model 3: Multivariate analysis excluding procedure because of confounding factor of cervical spine lesion

Model 4: Multivariate analysis excluding cervical spine lesion because of confounding factor of procedure

*OR* odds ratio, *CI* confidence interval, *Ref* reference, *PSL* prednisolone, *SAS* subaxial subluxation, *C1/2 fusion* atlantoaxial fusion, *OC fusion* occipito-cervical fusion

## Discussion

Patients with RA have been shown to experience a variety of complications after spine surgery more frequently than patients without RA [18, 27, 28]. This study was performed to detect the risk factors for complications in patients with RA who underwent all types of cervical spine surgeries. The prevalence of complications was 20.1% in the present study. We newly found that (1) short height, (2) high ASA-PS, (3) short disease duration of RA, and (4) long fusion procedures could be risk factors for perioperative complications. In addition, when focused on severe complications, (5) high-dose prednisolone administration, (6) existence of SAS, and (7) OC fusion and long fusion were suggested to be risk factors.

Prevalence of comorbidities and incidence of perioperative complication in patients with RA who underwent spine surgery were higher than patients without RA [18]. Higher class of ASA-PS was generally increased perioperative complications in all surgery including spine surgery [29–31]. The present study showed that the mean value of ASA-PS was significantly higher in patients with perioperative complications than in patients with no complications. On the other hand, there was no correlation between CCI and perioperative complications in the present study, although previous reports have suggested that the CCI could be useful to predict perioperative complications in spine surgery [32, 33]. The CCI represents the simple sum of comorbidities weighted based on the adjusted risk of mortality or resource use in the future. The ASA-PS are determined by anaesthesiologists considering not only types of comorbidity but also severity, which suggests that the ASA-PS could reflect the comorbidity status in more detail and more accurately than the CCI. The subtle differences between these two criteria concepts might make a difference in the strength of their relevance to complications depending on the underlying disease such as RA.

Both the existence of SAS and the long fusion procedures were detected as risk factors for severe perioperative complications after cervical spine surgery for patients with RA in the present study. Cervical spine lesions with RA generally start at the atlantoaxial joint, and they progress from AAS to VS and then SAS [34, 35]. Patients with SAS are expected to have progressive systemic joint damage [34, 36] as well as multiple comorbidities [37]. In addition to the risks associated with systemic conditions before surgery, operative procedures for SAS were also related to perioperative complications. Treatment for symptomatic SAS usually requires a long fusion procedure, which fixes many vertebrae and places a much greater burden on the patient’s body than laminoplasty or C1–2 fusion [3]. Thus, the presence of preoperative comorbidities and an increase in surgical burden may be associated with perioperative complications in patients with SAS lesions who underwent long fusion. Moreover, OC fusion was also detected as a risk factor for severe complications. OC fusion required longer surgery times than laminoplasty and C1-2 fusion. This result suggested that OC fusion placed a moderate surgical burden on the patients but not as much as long fusion. Otherwise, poor general condition or highly invasive OC fusion procedures might influence the incidence of severe complications but not mild/moderate complications.

The use of high-dose prednisolone was also detected as a risk factor for severe perioperative complications in the present study, although prednisolone use and its dose did not affect the incidence of perioperative complications including infection in patients with Crohn’s disease [38] or in patients with RA who underwent cervical spine and prosthesis surgery [39, 40]. Considering the background of the patients who were taking high-dose prednisolone for RA, these patients are expected to have high disease activity and a limited drug selection due to comorbidities. Administration of high-dose prednisolone might only identify a population prone to perioperative complications, and the increased risk for complications may not be an effect of the drug itself.

Physical constitution could affect perioperative complications and anesthesia management. In fact, obesity correlates with not only comorbidities in patients with RA [41] but also perioperative complications after many types of surgery [42–46]. However, there have been only a few reports that shorter patients were at higher risk for perioperative complications, which were shown in coronal and carotid endarterectomy [47]. Body size was suggested to have a direct impact on technical issues related to the surgery, such as limited access to the surgical field, since both BSA and height have been shown to correlate closely with the diameter of the common carotid artery [48]. Surgery in a small, limited field might affect certain kinds of surgical techniques, resulting in an increase in perioperative complications. However, it was difficult to substantiate this possibility with cervical spine surgery because there were no significant inverse correlations between short height and operation time or bleeding volume in the present study.

A shorter duration of RA in the patients with perioperative complications than in the patients without complications was shown in the present study. However, the mean disease duration of RA at the time of cervical spine surgery was 17.6 years in the complication group and 20.2 years in the no-complication group. It is difficult to determine the clinical meaning of this 2.5-year difference over such a long disease duration. A possible reason might be related to the features of cervical spine lesions in patients with RA. Most studies have reported that cervical spine lesions are a feature of longstanding rather than an early disease, which are generally apparent ten years into the natural history of RA [49, 50]. On the other hand, the extension of progression at cervical spine lesions has also been associated with the severity of peripheral radiographic joint damage [8, 51] as well as the number of erosive joints in the hand and foot [34, 36]. In addition, an increasing number of comorbidities correlated to poorer values for tender joint count and swollen joint count [37]. These findings suggested that patients with uncontrollable inflammatory arthritis in the systemic joint progressed to cervical spine lesions early (i.e., with a short disease duration) and tended to have more comorbidities, which resulted in increased perioperative complications after cervical spine surgery.

There were two limitations in the present study. First, there could have been heterogeneity in the background conditions over time. The patients enrolled in the present study were enrolled over the last two decades. During this 20-year period, the general condition of patients with RA and the pathophysiology of cervical spine lesions has changed, as pharmacological treatment for RA has changed dramatically after the emergence of biologics and JAK inhibitors. Considering that most patients used prednisolone and a few patients used biologics, the patient group in the present study seemed to be a distinct population with relatively progressed RA and some comorbidities. In addition, instruments for fixation have advanced or been modified year after year. These biases could have resulted in an underestimate or overestimate in the analysis. The second limitation was data deficiency. Physical examinations or questionnaires assessing disease activity or physiological function in the patients with RA could not be investigated. Anti-citrullinated protein antibodies, rheumatoid factor, or matrix metalloproteinase-3 were not detected in some cases. In addition, many patients did not have radiographs available except for the cervical spine. These data deficiencies limited the ability to directly investigate correlations of perioperative complications with disease activity or progression of RA, biomarker contributions, and the existence of systemic joint destruction.

## Conclusion

Low height, high ASA-PS, high-dose prednisolone use, and the progression of cervical spine lesions in early disease stages could be risk factors for perioperative complications. Surgical intervention should be appropriately considered when patients have symptomatic AAS to prevent the progression of cervical spine lesions, and this approach could also prevent further perioperative complications that might occur in later surgery for more advanced cervical spine lesions.

## Data Availability

The datasets used and/or analyzed during the current study are included in this published article or otherwise available from the corresponding author on reasonable request.

## Abbreviations

RA: Rheumatoid arthritis
ASA-PS: American Society of Anesthesiologists Physical Status
CCI: Charlson comorbidity index
CTCAE: Common Terminology Criteria for Adverse Event
AAS: Atlantoaxial subluxation
ADI: Atlantodental interval
VS: Vertical subluxation
SAS: Aubaxial subluxation
C1/2 fusion: Atlantoaxial fusion
OC fusion: Occipito-cervical fusion
SSI: Surgical site infection

## References

[1] Smolen JS, Aletaha D, McInnes IB. Rheumatoid arthritis. Lancet. 2016;388:2023–38. 10.1016/S0140-6736(16)30173-8 27156434

[2] Wick MC. Relationship between inflammation and joint destruction in early rheumatoid arthritis: a mathematical description. Ann Rheum Dis. 2004;63:848–52. 10.1136/ard.2003.015172 PMC1755049 15194582

[3] Gillick JL, Wainwright J, Das K. Rheumatoid arthritis and the cervical spine: a review on the role of surgery. Int J Rheumatol. 2015;2015:252456. 10.1155/2015/252456 PMC4553335 26351458

[4] Goodman SM, Mirza SZ, DiCarlo EF, Pearce-Fisher D, Zhang M, Mehta B, et al. Rheumatoid arthritis flares after Total hip and Total knee Arthroplasty: outcomes at one year. Arthritis Care Res. 2020;72:925–32. 10.1002/acr.24091 PMC7153968 31609524

[5] van Asselt KM. Outcome of cervical spine surgery in patients with rheumatoid arthritis. Ann Rheum Dis. 2001;60:448–52. 10.1136/ard.60.5.448 PMC1753638 11302865

[6] Burn E, Edwards CJ, Murray DW, Silman A, Cooper C, Arden NK, et al. The effect of rheumatoid arthritis on patient-reported outcomes following knee and hip replacement: evidence from routinely collected data. Rheumatology. 2019;58:1016–24. 10.1093/rheumatology/key409 PMC6532447 30608608

[7] Zhang T, Pope J. Cervical spine involvement in rheumatoid arthritis over time: results from a meta-analysis. Arthr Res Ther. 2015;17:148. 10.1186/s13075-015-0643-0 PMC4449959 26026719

[8] del Grande M, del Grande F, Carrino J, Bingham CO, Louie GH. Cervical spine involvement early in the course of rheumatoid arthritis. Semin Arthritis Rheum. 2014;43:738–44. 10.1016/j.semarthrit.2013.12.001 24444595

[9] Wolfs JFC, Kloppenburg M, Fehlings MG, van Tulder MW, Boers M, Peul WC. Neurologic outcome of surgical and conservative treatment of rheumatoid cervical spine subluxation: a systematic review. Arthritis Rheum. 2009;61:1743–52. 10.1002/art.25011 19950322

[10] Matsunaga S, Sakou T, Onishi T, Hayashi K, Taketomi E, Sunahara N, et al. Prognosis of patients with upper cervical lesions caused by rheumatoid arthritis: comparison of occipitocervical fusion between c1 laminectomy and nonsurgical management. Spine. 2003;28:1581–7. 12865848

[11] Tanaka N, Sakahashi H, Hirose K, Ishima T, Takahashi H, Ishii S. Results after 24 years of prophylactic surgery for rheumatoid atlantoaxial subluxation. J Bone Joint Surg. 2005;87:955–8. 10.1302/0301-620X.87B7.15862 15972910

[12] Krauss WE, Bledsoe JM, Clarke MJ, Nottmeier EW, Pichelmann MA. Rheumatoid arthritis of the Craniovertebral junction. Neurosurgery. 2010;66:83–95. 10.1227/01.NEU.0000365854.13997.B0 20173532

[13] Joaquim AF, Appenzeller S. Cervical spine involvement in rheumatoid arthritis — a systematic review. Autoimmun Rev. 2014;13:1195–202. 10.1016/j.autrev.2014.08.014 25151973

[14] Wasserman BR, Moskovich R, Razi AE. Rheumatoid arthritis of the cervical spine--clinical considerations. Bull NYU Hosp Joint Dis. 2011;69:136–48. 22035393

[15] Vu Nguyen H, Ludwig SC, Silber J, Gelb DE, Anderson PA, Frank L, et al. Rheumatoid arthritis of the cervical spine. Spine J. 2004;4:329–34. 10.1016/j.spinee.2003.10.006 15125859

[16] Mallory GW, Halasz SR, Clarke MJ. Advances in the treatment of cervical rheumatoid: less surgery and less morbidity. World J Orthoped. 2014;5:292–303. 10.5312/wjo.v5.i3.292 PMC4095022 25035832

[17] Horowitz JA, Puvanesarajah V, Jain A, Li XJ, Shimer AL, Shen FH, et al. Rheumatoid arthritis is associated with an increased risk of postoperative infection and revision surgery in elderly patients undergoing anterior cervical fusion. Spine. 2018;43:E1040–4. 10.1097/BRS.0000000000002614 29481378

[18] Bernstein DN, Kurucan E, Menga EN, Molinari RW, Rubery PT, Mesfin A. Comparison of adult spinal deformity patients with and without rheumatoid arthritis undergoing primary non-cervical spinal fusion surgery: a nationwide analysis of 52,818 patients. Spine J. 2018;18:1861–6. 10.1016/j.spinee.2018.03.020 29631060

[19] Takenaka S, Kashii M, Iwasaki M, Makino T, Sakai Y, Kaito T. Risk factor analysis of surgery-related complications in primary cervical spine surgery for degenerative diseases using a surgeon-maintained database. Bone Joint J. 2021;103-B:157–63. 10.1302/0301-620X.103B1.BJJ-2020-1226.R1 33380205

[20] Marques PM, Cacho-Rodrigues P, Ribeiro-Silva M, Linhares D, Negrão P, Pinto R, et al. Surgical management of cervical spine instability in rheumatoid arthritis patients. Acta Reumatol Port. 2015;40:34–9. 25340997

[21] Charlson ME, Pompei P, Ales KL, MacKenzie CR (1987). A new method of classifying prognostic comorbidity in longitudinal studies: development and validation. J Chronic Dis.

[22] Abouleish AE, Leib ML, Cohen NH (2015). ASA provides examples to each ASA physical status class. ASA Newslet.

[23] Keats AS, The ASA. Classification of physical status–a recapitulation. Anesthesiology. 1978;49:233–6. 10.1097/00000542-197810000-00001 697075

[24] National Cancer Institute: Common Terminology Criteria for Adverse Events Version 5. (2017). https://ctep.cancer.gov/protocolDevelopment/electronic_applications/ctc.htm#ctc_50. Accessed 28 Feb 2022.

[25] Arnett FC, Edworthy SM, Bloch DA, Mcshane DJ, Fries JF, Cooper NS (1988). The american rheumatism association 1987 revised criteria for the classification of rheumatoid arthritis. Arthritis Rheum.

[26] Aletaha D, Neogi T, Silman AJ, Funovits J, Felson DT, Bingham CO (2010). 2010 rheumatoid arthritis classification criteria: an American College of Rheumatology/European league against rheumatism collaborative initiative. Ann Rheum Dis.

[27] Crawford CH, Carreon LY, Djurasovic M, Glassman SD. Lumbar fusion outcomes in patients with rheumatoid arthritis. Eur Spine J. 2008;17:822–5. 10.1007/s00586-008-0610-4 PMC2518997 18228051

[28] Kang C-N, Kim C-W, Moon J-K. The outcomes of instrumented posterolateral lumbar fusion in patients with rheumatoid arthritis. Bone Joint J. 2016;98-B:102–8. 10.1302/0301-620X.98B1.36247 26733522

[29] Wolters U, Wolf T, Stützer H, Schröder T. ASA classification and perioperative variables as predictors of postoperative outcome. Br J Anaesth. 1996;77:217–22. 10.1093/bja/77.2.217 8881629

[30] Somani S, di Capua J, Kim JS, Phan K, Lee NJ, Kothari P, et al. ASA classification as a risk stratification tool in adult spinal deformity surgery: a study of 5805 patients. Glob Spine J. 2017;7:719–26. 10.1177/2192568217700106 PMC5721995 29238634

[31] Umekawa M, Takai K, Taniguchi M. Complications of spine surgery in elderly Japanese patients: implications for future of world population aging. Neurospine. 2019;16:780–8. 10.14245/ns.1938184.092 PMC6944995 31446683

[32] Whitmore RG, Stephen JH, Vernick C, Campbell PG, Yadla S, Ghobrial GM, et al. ASA grade and Charlson comorbidity index of spinal surgery patients: correlation with complications and societal costs. Spine J. 2014;14:31–8. 10.1016/j.spinee.2013.03.011 23602377

[33] Ranson WA, Neifert SN, Cheung ZB, Mikhail CM, Caridi JM, Cho SK. Predicting in-hospital complications after anterior cervical discectomy and fusion: a comparison of the Elixhauser and Charlson comorbidity indices. World Neurosurg. 2020;134:e487–96. 10.1016/j.wneu.2019.10.102 31669536

[34] Oda T, Fujiwara K, Yonenobu K, Azuma B, Ochi T. Natural course of cervical spine lesions in rheumatoid arthritis. Spine. 1995;20:1128–35. 10.1097/00007632-199505150-00004 7638655

[35] Paimela L, Laasonen L, Kankaanpää E, Leirisalo-Repo M. Progression of cervical spine changes in patients with early rheumatoid arthritis. J Rheumatol. 1997;24:1280–4. 9228125

[36] Ahn JK, Hwang J-W, Oh J-M, Lee J, Lee YS, Jeon CH, et al. Risk factors for development and progression of atlantoaxial subluxation in Korean patients with rheumatoid arthritis. Rheumatol Int. 2011;31:1363–8. 10.1007/s00296-010-1437-y 20422194

[37] Luque Ramos A, Redeker I, Hoffmann F, Callhoff J, Zink A, Albrecht K. Comorbidities in patients with rheumatoid arthritis and their association with patient-reported outcomes: results of claims data linked to questionnaire survey. J Rheumatol. 2019;46:564–71. 10.3899/jrheum.180668 30647170

[38] Bruewer M, Utech M, Rijcken EJM, Anthoni C, Laukoetter MG, Kersting S, et al. Preoperative steroid administration: effect on morbidity among patients undergoing intestinal bowel resection for Crohńs disease. World J Surg. 2003;27:1306–10. 10.1007/s00268-003-6972-1 14716499

[39] Koyama K, Ohba T, Ebata S, Haro H. Postoperative surgical infection after spinal surgery in rheumatoid arthritis. Orthopedics. 2016;39:e430–3. 10.3928/01477447-20160404-05 27064779

[40] Okita S, Ishikawa H, Abe A, Ito S, Nakazono K, Murasawa A, et al. Risk factors of postoperative delayed wound healing in patients with rheumatoid arthritis treated with a biological agent. Mod Rheumatol. 2021;31:587–92. 10.1080/14397595.2020.1790138 32613884

[41] de Resende Guimarães MFB, Rodrigues CEM, Gomes KWP, Machado CJ, Brenol CV, Krampe SF, et al. High prevalence of obesity in rheumatoid arthritis patients: association with disease activity, hypertension, dyslipidemia and diabetes, a multi-center study. Adv Rheumatol. 2019;59:44. 10.1186/s42358-019-0089-1 31619287

[42] Dindo D, Muller MK, Weber M, Clavien P-A. Obesity in general elective surgery. Lancet (London, England). 2003;361:2032–5. 10.1016/S0140-6736(03)13640-9 12814714

[43] Houdek MT, Wagner ER, Watts CD, Osmon DR, Hanssen AD, Lewallen DG, et al. Morbid obesity: a significant risk factor for failure of two-stage revision total hip arthroplasty for infection. J Bone Joint Surg Am. 2015;97:326–32. 10.2106/JBJS.N.00515 25695985

[44] Jiang J, Teng Y, Fan Z, Khan S, Xia Y. Does obesity affect the surgical outcome and complication rates of spinal surgery? A meta-analysis. Clin Orthop Relat Res. 2014;472:968–75. 10.1007/s11999-013-3346-3 PMC3916601 24146361

[45] Arance García M, Docobo Durántez F, Conde Guzmán C, Pérez Torres MC, Martín-Gil Parra R, Fernández Jiménez PE. Is obesity a risk factor for complications, hospital admissions, and surgical cancellations in ambulatory surgery? Rev Esp Anestesiol Reanim. 2015;62:125–32. 10.1016/j.redar.2014.03.016 25048995

[46] Gurunathan U, Myles PS. Limitations of body mass index as an obesity measure of perioperative risk. Br J Anaesth. 2016;116:319–21. 10.1093/bja/aev541 26865129

[47] Messé SR, Kasner SE, Mehta Z, Warlow CP, Rothwell PM, European Carotid Surgery Trialists. Effect of body size on operative risk of carotid endarterectomy. J Neurol Neurosurg Psychiatry. 2004;75:1759–61. 10.1136/jnnp.2003.030486 PMC1738869 15548500

[48] Mortensen JD, Talbot S, Burkart JA. Cross-sectional internal diameters of human cervical and femoral blood vessels: relationship to subject’s sex, age, body size. Anat Rec. 1990;226:115–24. 10.1002/ar.1092260114 2297079

[49] Hagenow A, Seifert J, Zeissig A, Conrad K, Kleymann A, Aringer M. Relevant incidence of cervical arthritis in patients with erosive seropositive rheumatoid arthritis even today. Clin Exp Rheumatol. 2013;31:213–8. 23295192

[50] Dreyer SJ, Boden SD. Natural history of rheumatoid arthritis of the cervical spine. Clin Orthop Relat Res. 1999:98–106. 10.1097/00003086-199909000-00013 10627723

[51] Oláh C, Kardos Z, Kostyál L, Hodosi K, Tamási L, Bereczki D, et al. Assessment of cervical spine involvement in rheumatoid arthritis patients in the era of biologics: a real-life, cross-sectional MRI study. Rheumatol Int. 2020;40:915–21. 10.1007/s00296-020-04549-w 32180009

